# 异孔共价有机框架气相色谱固定相的制备与应用

**DOI:** 10.3724/SP.J.1123.2026.01035

**Published:** 2026-10-08

**Authors:** Zhiquan LI, Zirui RAO, Hailong QIAN, Xiuping YAN

**Affiliations:** 江南大学食品学院分析食品安全学研究所，江苏 无锡 214122; Institute of Analytical Food Safety，School of Food Science and Technology，Jiangnan University，Wuxi 214122，China

**Keywords:** 气相色谱, 固定相, Kagome拓扑结构, 异孔共价有机框架, 异构体分离, gas chromatography （GC）, stationary phase, Kagome topological structure, heteropore covalent organic frameworks, isomer separation

## Abstract

共价有机框架（COFs）因其结构可设计、功能基团易修饰和孔径尺寸可调等特点，在分离科学中展现出显著潜力，是一种良好的色谱分离介质。本研究提出一种基于拓扑结构调控COF孔径的策略，旨在构建具有不同孔径分布的异孔COF，提升其在气相色谱分离异构体中的性能。本工作选用具有*D*_2h_对称性的四胺单体4，4′，4″，4‴-（乙烯-1，1，2，2-四基）四苯胺（ETTA）与具有*C*_2_对称性的线性连接体对苯二甲醛（TPA），通过亚胺缩合反应构建了一种具有Kagome拓扑的异孔COF（命名为ET-COF）。氮气吸附-脱附实验证明ET-COF呈现出明显的双孔结构，包括孔径分布为1.1 nm的微孔和2.89 nm的介孔，且比表面积达724 m^2^/g，孔体积为1.26 cm^3^/g，丰富的孔结构与高比表面积赋予其多重尺寸排阻效应；通过原位生长法制备了ET-COF键合毛细管色谱柱，色谱性能评价结果表明，ET-COF丰富的孔结构赋予该柱较优的分离性能，对一系列同系物（正构烷烃、烷基苯、直链脂肪酸酯）以及3种C9芳烃异构体均实现了基线分离，分离度范围为2.0~15.3。本工作研究了异孔结构（微孔择形与介孔传质协同）对分离性能的影响，结果表明该结构显著提升了固定相分离异构体的能力，为异孔COFs在色谱固定相中的应用提供了新的设计思路，并进一步推动了COFs在分离分析领域的实用化进程。

## Abstract (en)

Chromatographic separation stands as a cornerstone technique in modern analytical chemistry， playing an indispensable role in critical areas such as chemical purification， environmental monitoring， and pharmaceutical analysis. With the increasing complexity of analytical samples， particularly regarding the efficient separation of structurally similar isomers， higher requirements have been imposed on chromatographic stationary phases. Traditional porous materials such as zeolites and silica show limited tunability in pore size distribution and surface chemistry， making it difficult to meet the urgent need for high-resolution and rapid separation of complex mixtures. Therefore， the exploration of novel ordered porous materials with precisely tunable pore structures and functionalities has become a frontier research direction in separation science. Covalent organic frameworks （COFs）， as a category of crystalline porous polymers， possess great application potential in separation science. Their prominent advantages mainly include designable periodic frameworks， easily modified functional groups， and precisely tunable pore sizes. These intrinsic merits render COFs promising candidates for next-generation chromatographic separation media. In this work， a novel strategy is proposed to regulate the pore structure of COFs via rational topological design， aiming to improve their gas chromatographic performance toward challenging isomer separation. This is achieved by constructing heteroporous COFs that integrate distinct pore size distributions within a single， ordered framework. To realize this strategy， a heteroporous COF designated as ET-COF was successfully constructed. It was synthesized via a reversible imine condensation reaction between a *D*_2h_-symmetric tetraamine monomer， 4，4′，4″，4′′′ -（ethene-1，1，2，2-tetrayl）tetraaniline （ETTA）， and a *C*_2_-symmetric linear dialdehyde linker， terephthalaldehyde （TPA）. This assembly affords a two-dimensional framework with a Kagome topology. The porous structures of material were rigorously characterized. It has been demonstrated through nitrogen adsorption-desorption experiments that ET-COF exhibits a distinct dual-pore structure， including triangular micropores of approximately 1.1 nm and hexagonal mesopores of 2.89 nm， with a specific surface area of 724 m^2^/g and a pore volume of 1.26 cm^3^/g. The rich pore structure and high specific surface area confer multiple size-exclusion effects. By virtue of these structural merits， an ET-COF-bonded capillary chromatographic column was further prepared via a facile in-situ growth method， which enables stable and uniform strong chemical bonding of the COF layer to the inner wall of a fused-silica capillary. Finally， the chromatographic performance of this column was systematically evaluated. The heteroporous ET-COF column demonstrated excellent separation performance across a wide range of test analytes. Baseline separation was successfully achieved for diverse mixtures， including homologous *n*-alkanes （C5-C10）， alkylbenzenes （from benzene to butylbenzene）， *n*-straight-chain fatty acid esters， and critically a set of C9 aromatic hydrocarbon isomers （*n*-propylbenzene， isopropylbenzene， and mesitylene）， with resolution values ranging from 2.0 to 15.3， suggesting outstanding separation efficiency. The improved chromatographic behavior can be rationalized by the uniquely hierarchical pore structure originating from rational topological design. The triangular micropores afford a confined microenvironment that imposes prominent size- and shape-selectivity， enabling discrimination of isomers with subtle differences in molecular configuration. Meanwhile， the hexagonal mesopores act as rapid transport pathways to promote fast mass transfer and avoid pore blocking. Such a synergistic effect of selective micropores and high-flux mesopores within one crystalline framework represents a distinct merit of the heteroporous topology. In summary， this work establishes a feasible topological strategy for constructing high-performance COF-based stationary phases and investigates the impact of hierarchical pore structures （synergistic effects of microporous shape selectivity and mesoporous mass transfer） on separation performance. By rational topological assembly， precise pore engineering and integration of hierarchical channels are readily realized， which greatly boost the separation selectivity toward challenging isomers. This study not only develops a promising heteroporous COF stationary phase for gas chromatography， but also provides a new design paradigm for extending the application of COFs in analytical separation science.

提高色谱分离能力一直是分析化学与分离科学的核心目标之一。在气相色谱中，固定相的性能直接决定着分离的选择性、效率和分辨率，因此，开发具有优异分辨能力和稳定性的新型固定相是该领域持续的研究前沿^［1，2］^。近年来，多孔晶体材料因其有序的孔道结构和可调控的物理化学性质，在色谱固定相设计中展现出显著潜力。其中，共价有机框架材料（COFs）作为一种由轻质元素通过强共价键连接而成的晶态多孔聚合物，具有高比表面积、结构可精确设计、孔道尺寸可调、热稳定性和化学稳定性良好等突出特点，在色谱分离领域展现出巨大潜力^［3-6］^。

目前，将COFs用作气相色谱固定相的研究主要围绕两大策略展开：一是通过对构筑单元进行功能化修饰，引入特定官能团，以调节孔道表面的化学环境和相互作用力，从而改善对目标物的吸附选择性^［4，7-9］^；二是通过拓扑结构设计，从几何层面调控孔道的形状、尺寸和空间分布，实现对分子尺寸和形状的筛分效应^［10，11］^。后者通常借助具有不同对称性的构筑单元来实现，例如，*C*_3_对称的单体与*C*_2_对称的线性连接体可聚合形成六方晶系的孔道结构。然而，尽管拓扑设计策略多样，目前所报道的绝大多数二维COFs仅包含单一类型、单一尺寸的孔道（通常为大于1 nm的均孔）^［12，13］^，这导致其在分离分子尺寸较小、结构差异细微的异构体时，往往缺乏足够的选择性差异^［14］^。虽然三维COFs能提供更交叉贯通的多维孔道体系，但其合成所需单体制备复杂、结晶度控制困难，大大限制了其实际应用^［15，16］^。合适的孔径可以增强主体与客体分子之间的相互作用^［17］^，对材料的孔径进行合理调控有望提升分离能力。因此，在二维COFs框架中引入多级孔结构，被视为改善小分子异构体分离性能的一个富有前景但尚未充分探索的方向。

异孔共价有机框架（heteropore COFs）正是在此背景下受到关注的一类材料，其特指在于同一晶格内周期性排布着不同尺寸或形状的孔道的COFs^［18-20］^。2014年，Zhao等^［21］^报道了由*D*_2h_对称的四胺单体与*C*_2_对称的线性醛单体缩合形成的异孔COF，其结构中同时存在三角形微孔和六边形大孔。这一突破性工作揭示了通过精确设计单体对称性与连接方式，可以打破传统均孔结构的限制。2016年，Thomas等^［22］^发展了“位点特异性生长”策略，通过锁定单体反应位点，成功合成了一系列结晶度高、结构规整的六角星形双孔COFs，进一步推动了异孔COFs的可控合成。2023年，Feng等^［23］^通过降低构筑块对称性的几何策略，成功合成了基于萘与薁的异构双孔二维COFs，其中薁基COF-Az表现出极窄的带隙（1.37 eV）和优异的NO₂传感性能，为调控异构双孔COFs的性质及研究其结构‑性能关系提供了新思路。2024年，Ma等^［24］^报道了一种三氟甲基功能化的异孔COF，并将其用作气相色谱固定相，成功分离了几种位置异构体，初步证明了异孔结构在色谱分离中的实用性。理论上，异孔结构能够通过对微孔和介孔/大孔的尺寸、形状及表面化学进行一体化设计，实现对不同尺寸范围分子的分级筛分与吸附，并可能通过微孔的“择形选择”与介孔的“快速传输”协同，优化分离动力学过程^［25］^。因此，异孔COFs的特殊性质赋予其在分离领域的潜力，有望实现不同尺寸异构体的分离。然而，目前异孔COFs作为色谱固定相的研究仍处于初步阶段，相关研究报道较少。

本研究制备了具有Kagome拓扑结构的异孔COF作为色谱固定相，以研究拓扑结构对异构体气相色谱分离的影响。以*D*_2h_对称性的4，4′，4″，4‴-（乙烯-1，1，2，2-四基）四苯胺（ETTA）与*C*_2_对称性的对苯二甲醛（TPA）反应，构建了兼具三角形微孔和六边形介孔的异孔COF（命名为ET-COF）。在系统表征材料结构的基础上，通过原位生长法制备ET-COF键合毛细管柱，系统评估了该固定相对一系列同系物（正构烷烃、烷基苯、直链脂肪酸酯）以及3种C9芳烃异构体的分离性能。基于上述研究，本工作不仅为高性能COFs色谱固定相的开发提供了新的拓扑设计思路，也为从多孔材料结构角度深入解析色谱分离过程奠定了基础。

## 1 实验部分

### 1.1 试剂与仪器

所有实验所用试剂纯度均在分析纯及以上，无需额外纯化即可使用。超纯水购自杭州娃哈哈集团有限公司（浙江杭州）；ETTA和TPA购自上海楷树化学科技有限公司；3-氨丙基三乙氧基硅烷（APTES）购自上海泰坦科技股份有限公司，四氢呋喃、冰乙酸、1，4-二氧六环、均三甲苯、氢氧化钠、盐酸、乙醇、甲醇购自国药集团化学试剂有限公司（上海）。熔融石英毛细管（内径320 μm）购自永年孟锐仪器科技有限公司（河北邯郸）。

D2 PHASER粉末X射线衍射仪（PXRD，德国Bruker AXS公司），NEXUS傅里叶变换红外光谱仪（FT-IR，美国Nicolet公司），TGA2热重分析仪（TGA，美国梅特勒-托利多公司），SU8100扫描电子显微镜（SEM，日本株式会社日立高新技术公司），Autosorb-iQ氮气吸附分析仪（美国Quantachrome公司），GC-2030气相色谱仪（日本Shimadzu公司）。

### 1.2 ET-COF的合成

ET-COF的合成参照文献［^21^］并作适当改进：首先准确称取ETTA（30 mg，0.076 4 mmol）和TPA（20.5 mg，0.153 mmol）置于35 mL Schlenk反应管中，加入1，4-二氧六环-均三甲苯（1:1，体积比，2 mL）的混合溶液，经超声处理10 min，使其分散均匀。随后，缓慢向混合溶液中加入0.2 mL 6 mol/L乙酸水溶液作为催化剂，继续超声5 min。随后进行3次严格的冷冻-抽真空-解冻循环脱气处理，最终在惰性气氛保护下完成密封操作，随后于120 ℃进行3天的溶剂热反应。反应结束后经离心获得橙红色沉淀物并使用四氢呋喃进行多次洗涤以去除未反应单体及残留溶剂，产物置于60 ℃真空干燥箱中真空干燥过夜，得到橙色ET-COF粉末。ET-COF的合成示意图见图1。

**图1:**
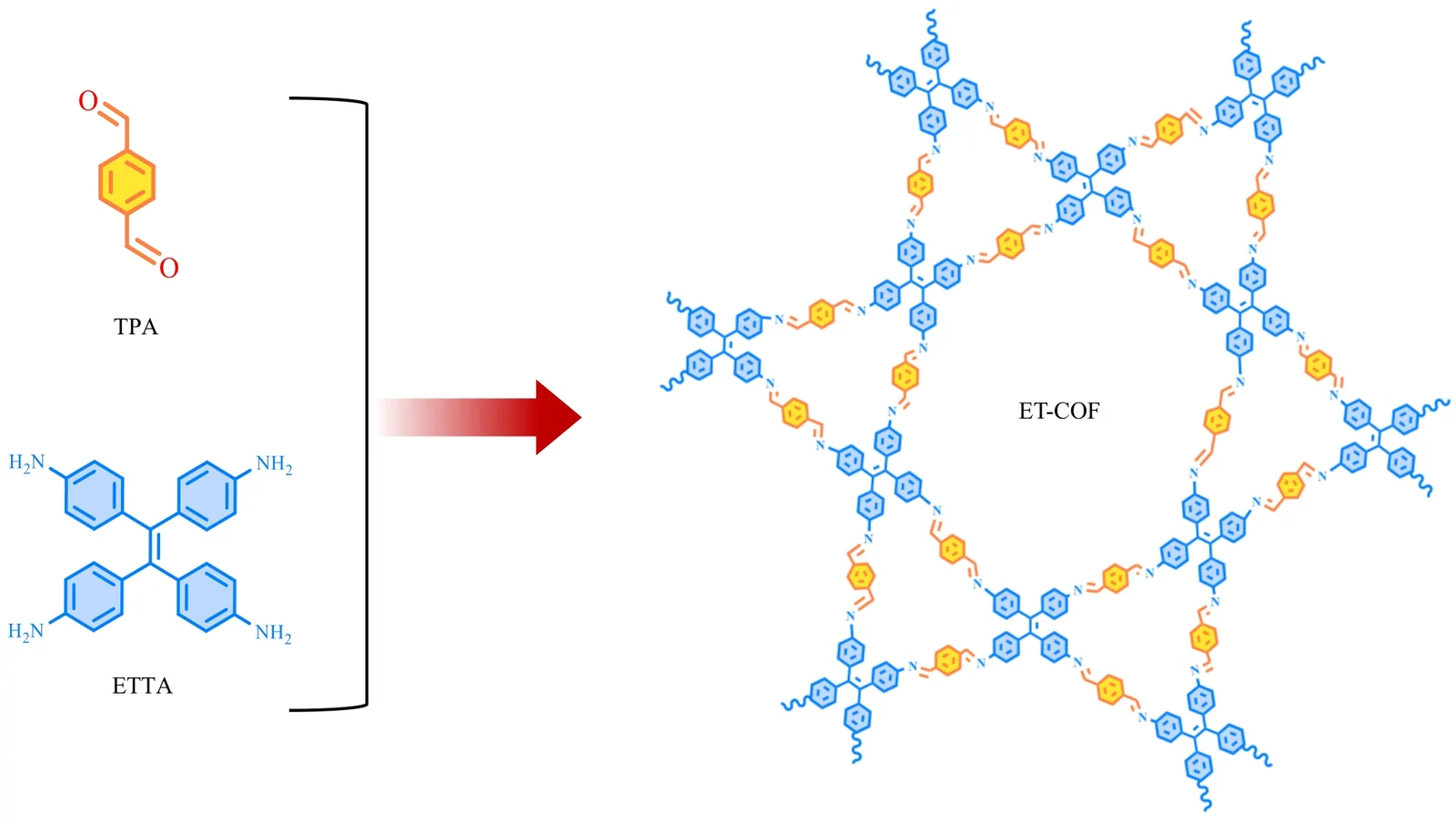
ET-COF的合成示意图

### 1.3 毛细管柱制备

#### 1.3.1 毛细管柱预处理

在制备毛细管柱之前先对熔融石英毛细管柱（15 m×0.32 mm）进行预处理：使用1 mol/L NaOH溶液注入毛细管柱并蚀刻2 h，纯水冲洗30 min至pH为中性，将0.1 mol/L盐酸溶液注入并保持2 h，纯水冲洗至流出液pH为中性，再用甲醇清洗30 min^［6］^。然后，用APTES-甲醇（1:1，体积比）溶液填充预处理的毛细管柱。将毛细管两端用小硅橡胶密封后，将毛细管置于70 ℃的水浴锅中，水浴12 h。最后，用甲醇冲洗APTES修饰的毛细管以去除游离APTES的残余物，并在120 ℃下用N_2_干燥老化2 h，得到APTES改性毛细管。

#### 1.3.2 ET-COF键合毛细管柱制备

准确称取8 mg ETTA和5.5 mg TPA单体，以1，4-二氧六环-均三甲苯（1:1，体积比，2 mL）为溶剂，并加入200 μL 6 mol/L乙酸水溶液作为催化剂，超声5 min使其混合均匀，使用注射器将其注入APTES改性毛细管柱内，保持毛细管柱两端密封，在120 ℃条件下反应72 h。反应完成后，使用乙醇冲洗毛细管柱，并使用N_2_吹干。在使用之前，将所制备毛细管柱在气相色谱仪上进行老化，老化程序如下：从60 ℃以5 ℃/min的升温速率升温至180 ℃，并在此条件下保持60 min，流速为1 mL/min。

### 1.4 气相色谱条件

气相色谱分离实验均在配备FID的岛津GC-2030气相色谱仪上完成。实验采用N_2_（99.999%）作为载气，载气流速以实际分析物标注为准，采用分流模式进样，分流比为80:1。实验数据均由LabSolutions软件采集并处理。实验中所使用的色谱柱为ET-COF键合毛细管柱（15 m×0.32 mm）。

## 2 结果与讨论

### 2.1 材料粉末表征

本研究选择具有*D*_2h_对称性的氨基单体ETTA和具有*C*_2_对称性的线性连接体醛基单体TPA作为构筑单元，利用溶剂热法通过席夫碱反应合成ET-COF。根据文献［^21^］，所得ET-COF具有六边形孔与三角形孔交替周期性排列的孔道结构。

ET-COF的粉末X射线衍射图谱中出现在2.74°的特征衍射峰证实了其有序的二维晶体结构，表明所制备材料具有良好的结晶性（图2a）。使用红外光谱对材料反应过程中化学结构的变化进行表征。在反应之后，TPA单体在1 683 cm^-1^处的C=O特征吸收峰的强度明显减弱，ETTA单体在3 200～3 500 cm^-1^处的氨基特征吸收峰消失，而所制备的ET-COF在1 619 cm^-1^处出现新的特征吸收峰，对应醛基和氨基反应生成的C=N伸缩振动吸收峰，证明席夫碱反应的发生（图2b）。进一步通过扫描电镜对所制备的COF进行形貌表征。如图2c、2d所示，制备的ET-COF呈现出非常规则的片状形貌。

**图2:**
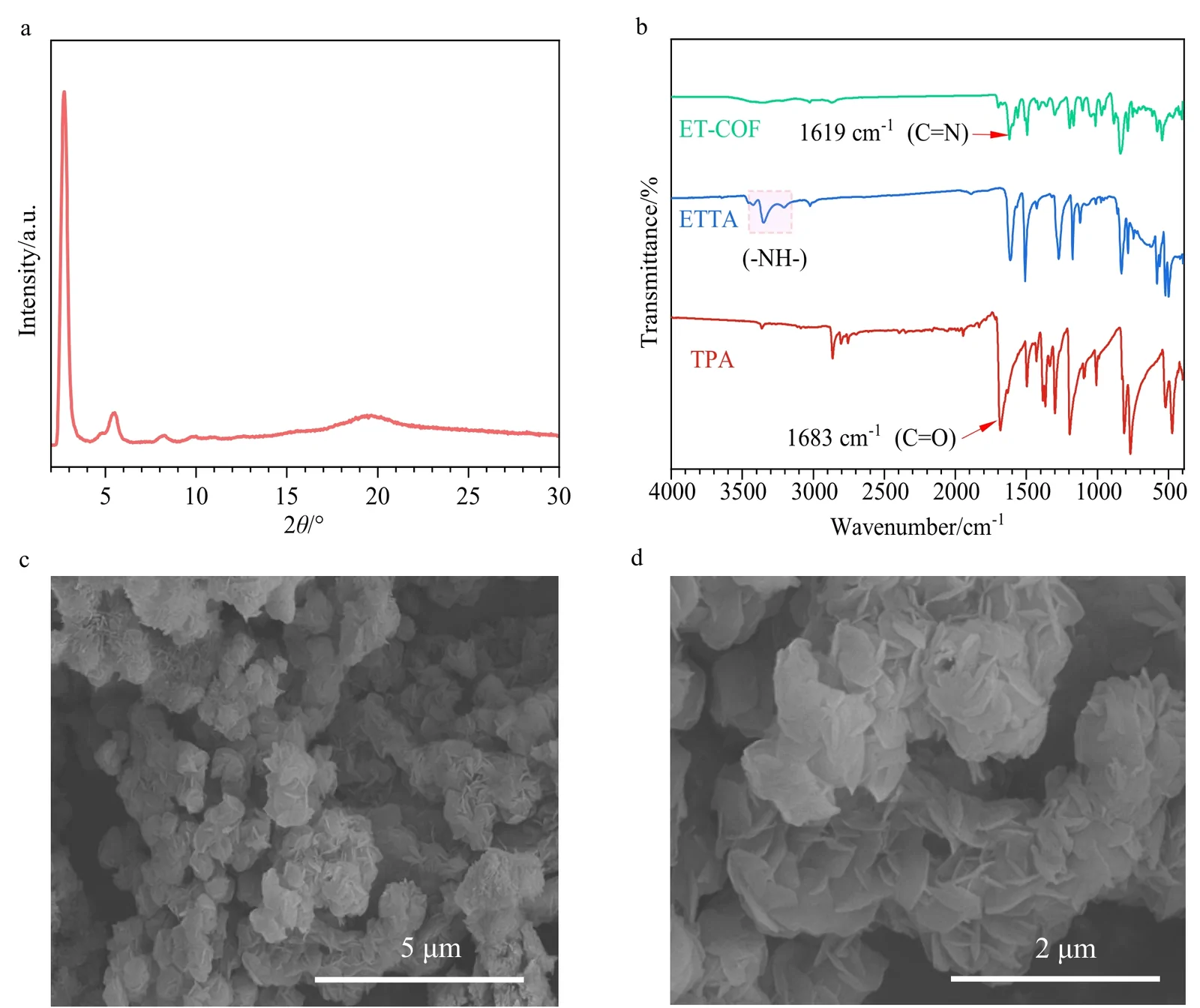
ET-COF的（a）PXRD衍射图、（b）红外光谱图和（c，d）扫描电镜图

用自动吸附分析仪测定气体吸附等温线。N_2_等温线用液氮浴（77 K），所使用的N_2_气体为超高压等级。测定结束后得到的吸附-解吸等温线用Quantachrome-AsiQwin软件分析，根据不同模型的拟合度选取最佳模型作为孔径和比表面积计算模型。图3a展示了ET-COF的氮气吸附-解吸等温线分析，整个曲线表现出Ⅰ型和Ⅳ型吸附等温线，表明该材料具有微孔和介孔特性；运用非局部密度泛函理论（NLDFT）模型得出其同时具有1.1 nm和2.89 nm的孔径（图3b），这一结果再次证实了双孔COF的形成^［26］^。依据Brunauer-Emmett-Teller（BET）模型，计算得出比表面积为724 m^2^/g，同时测得孔体积为1.26 cm^3^/g（*p*/*p*_0_=0.99）。多种孔隙结构的引入丰富了COF结构的复杂性，同时增强了与小动力学分子直径目标物之间的尺寸匹配作用力。

**图3:**
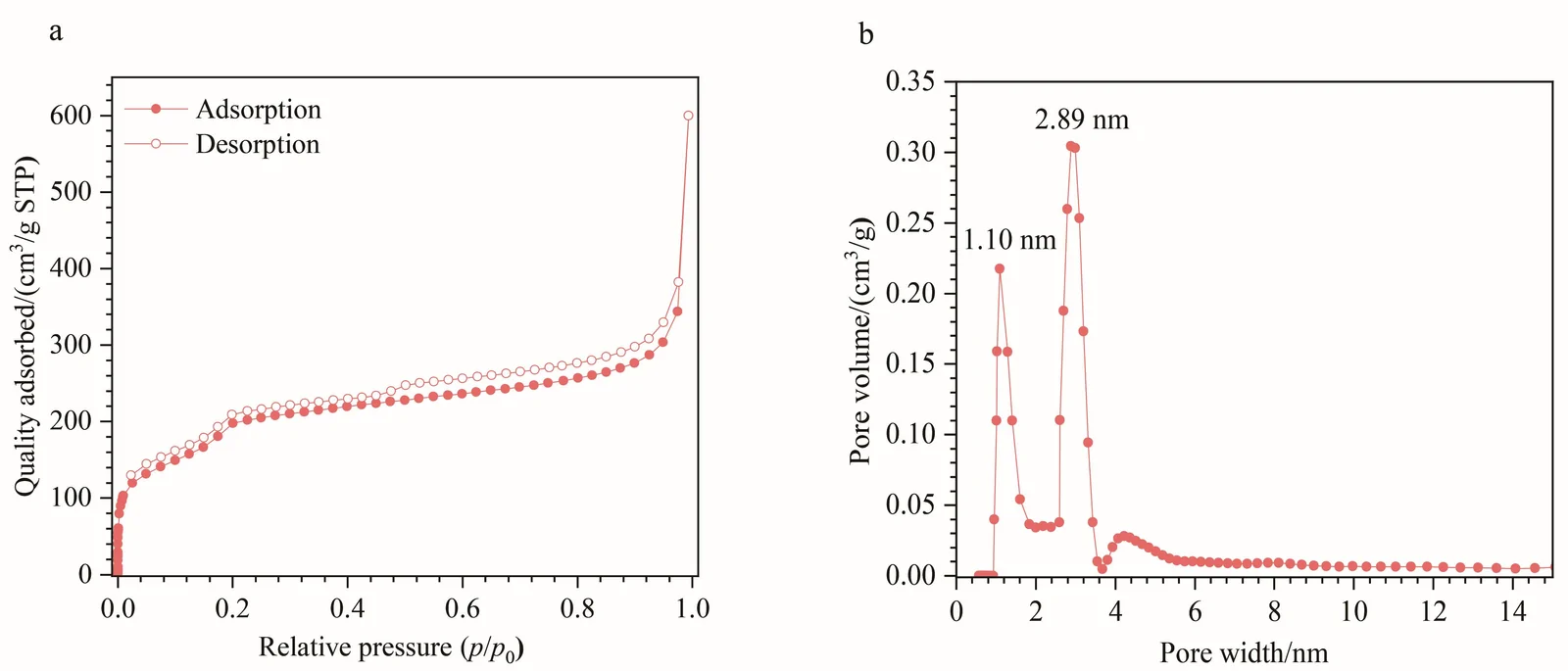
ET-COF的（a）氮气吸附-解吸等温线和（b）孔径分布

热稳定性良好是材料作为气相色谱固定相使用的前提，ET-COF的热稳定性由热重分析仪进行评估。在N_2_气氛下，温度从30 ℃上升到800 ℃，升温速率为10 ℃/min。如图4所示，其第一阶段的失重主要源于结合水分子的蒸发和残留有机溶剂去除过程，而第二阶段的失重则是由COF共价骨架的热分解或断裂过程所引发。总体来看，ET-COF在温度达到490 ℃时，其失重率仅为15%，显示出优异的热稳定性。当温度上升到490～800 ℃时，ET-COF的有序骨架结构逐步崩解，其残余质量不断下降。该结果证明ET-COF材料能较好地满足后续作为固定相时对热稳定性的要求。

**图4:**
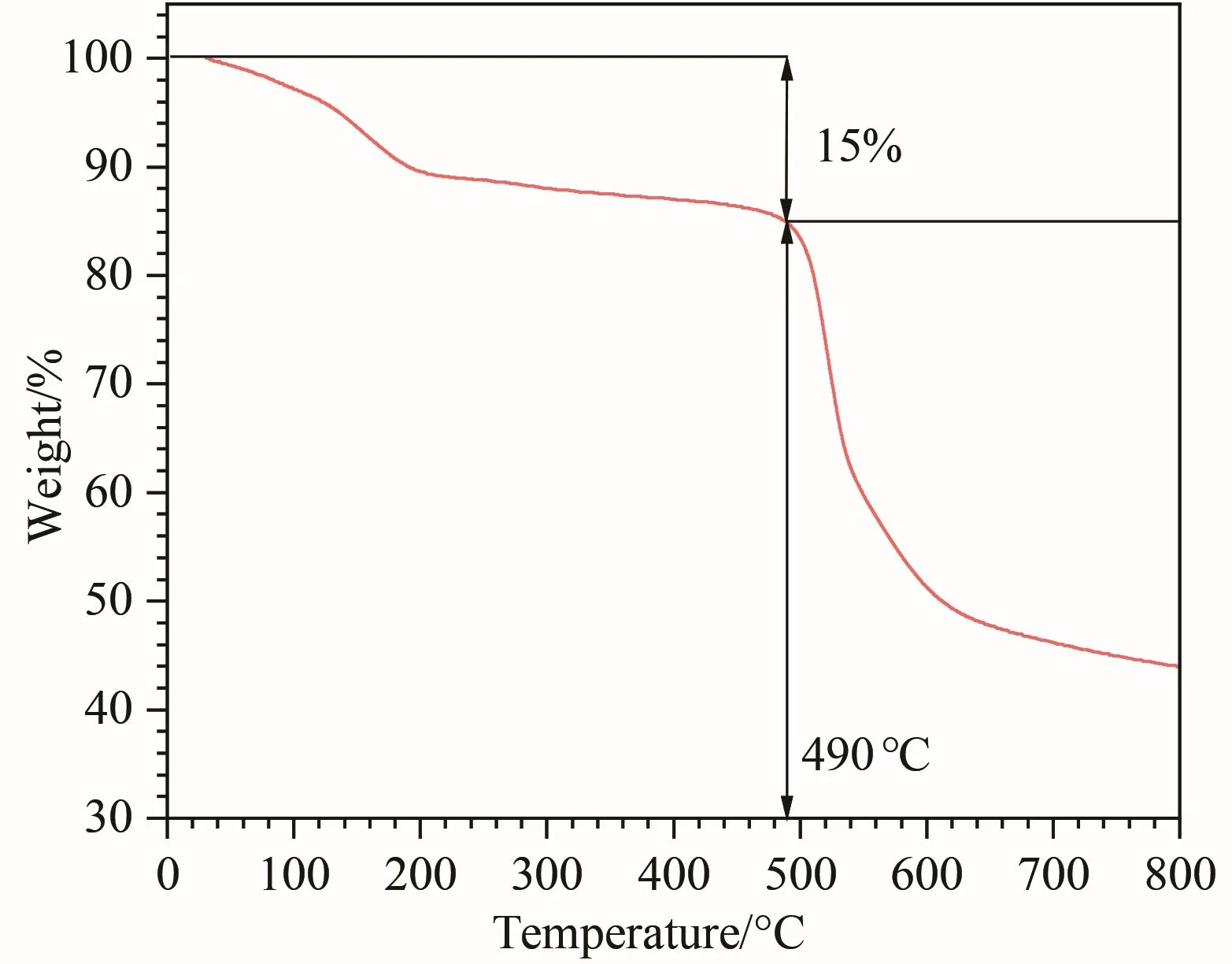
ET-COF的热重分析曲线

### 2.2 共价有机框架毛细管柱的表征

毛细管柱采用原位生长法制备，COFs材料与毛细管柱内壁通过共价键连接，有利于制备稳定性良好的色谱柱。采用扫描电镜对毛细管柱内壁进行表征，未进行共价修饰的裸毛细管柱内壁光滑（图5a）；使用COFs修饰后，ET-COF键合毛细管柱内壁皆有材料均匀生长且呈现片层状结构，与前文中粉末材料形貌特征基本一致，证明COFs键合毛细管柱的成功制备（图5b~d）。

**图5:**
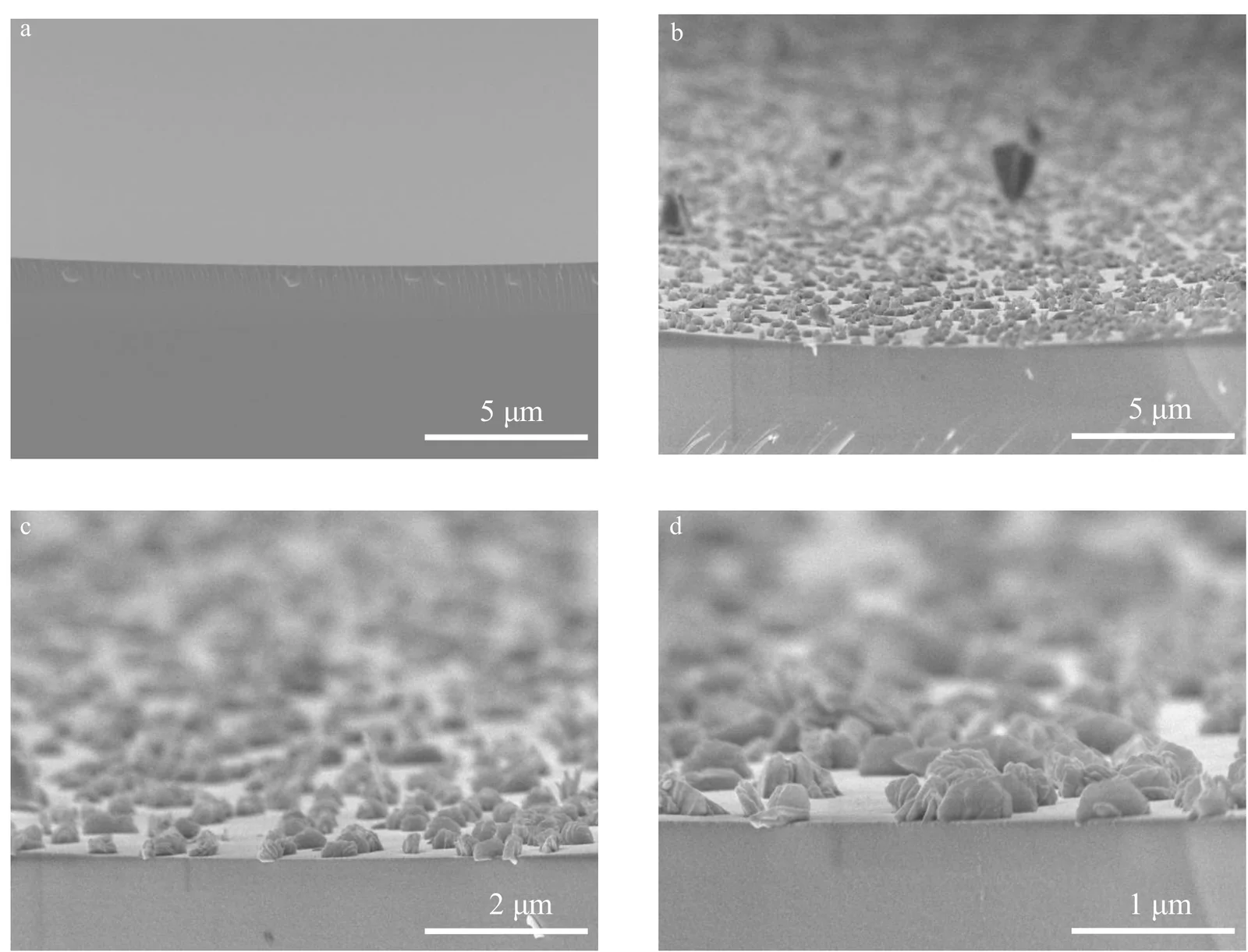
（a）裸毛细管柱和（b~d）ET-COF的扫描电镜图

对所制备毛细管柱内材料的结晶性进行表征，PXRD结果可证明毛细管柱内壁材料具有与溶剂热法合成材料一致的结晶性（图6a）；为了进一步证明ET-COF键合的毛细管柱的成功制备，采用红外光谱对所研磨的COF毛细管柱粉末的结构进行了表征。如图6b所示，毛细管柱出现了氨基和醛基反应生成的亚胺（C=N）伸缩振动吸收峰（1 620 cm^-1^），而在裸柱上并未发现亚胺（C=N）伸缩振动吸收峰，表明COF材料在毛细管柱内壁成功生长。

**图6:**
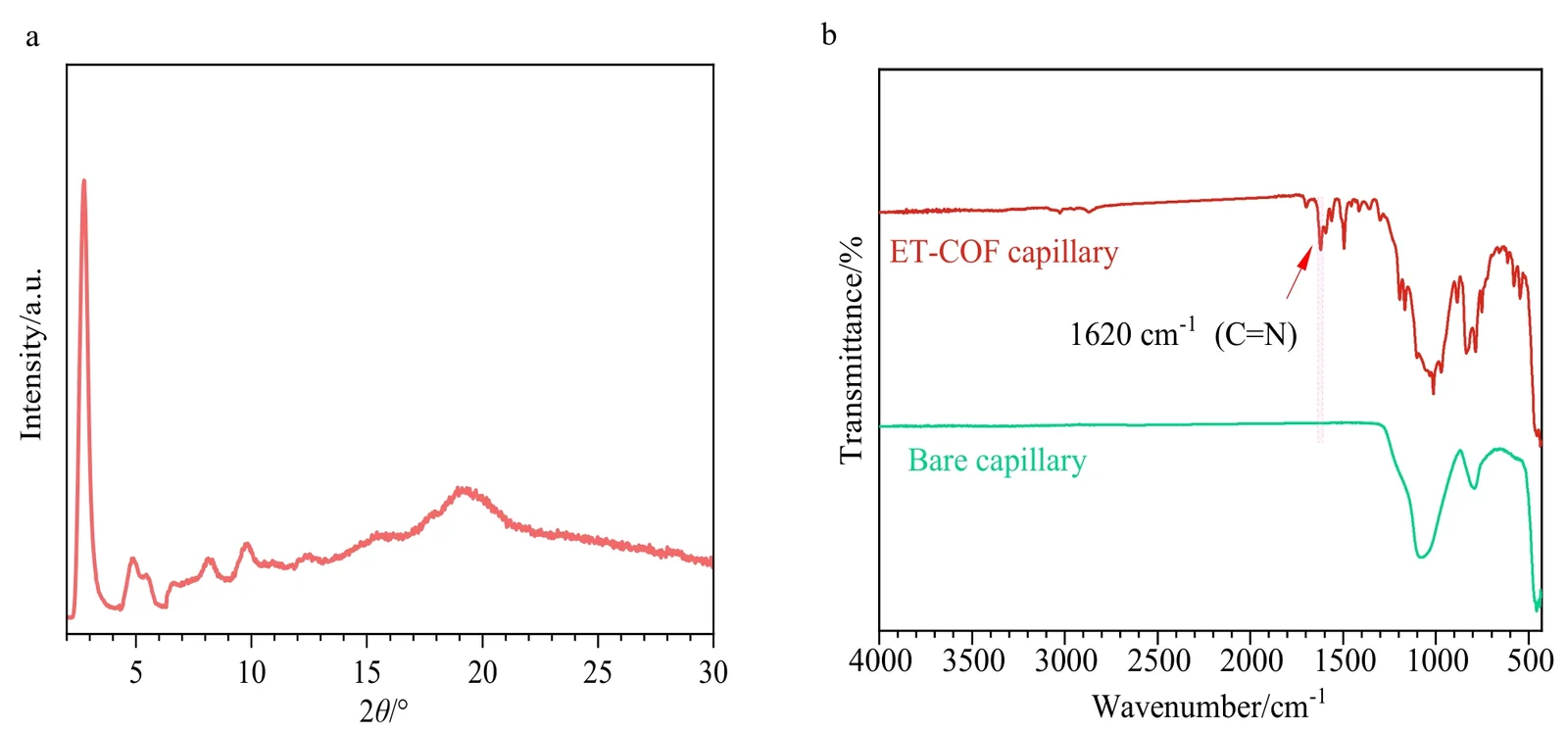
（a）键合在毛细管柱内壁上的ET-COF的PXRD图；（b）裸毛细管柱和ET-COF键合毛细管柱的红外光谱图

### 2.3 毛细管柱的分离性能

为了考察ET-COF键合色谱柱的分离能力，将所制备色谱柱应用于同系物的分离。正构烷烃和烷基苯（芳香烃混合物）在石油化工行业具有重要的意义，本研究测试了6个正构烷烃（C5～C10）混合物以及5个烷基苯混合物（苯、甲苯、乙苯、正丙苯、正丁苯）在ET-COF毛细管柱上的分离效果。如图7a、7b所示，这两组混合物中的所有组分都可以在该柱上实现基线分离。在程序升温条件下，正构烷烃（C5～C10）均可很好地被拆分，且基线平稳，同时分离时间不超过5 min，分析物在色谱柱上的洗脱顺序与沸点增加顺序一致，理论塔板数为15 105～41 052 plates/m，表明ET-COF毛细管柱分离正构烷烃效果很好（图7a）。同时，在程序升温条件下5 min内实现烷基苯（苯、甲苯、乙苯、正丙苯和正丁苯）的基线分离（图7b），证明所制备的ET-COF色谱柱具有普适性（相关参数见表1）。酯类在食品中是重要的呈味物质，将所制备的ET-COF色谱柱用于酯类混合物的分离。在程序升温条件下可在4 min内实现丙酸乙酯、丁酸乙酯、戊酸乙酯、己酸乙酯、庚酸乙酯、辛酸乙酯的酯类混合物的基线分离（图7c）。

**图7:**
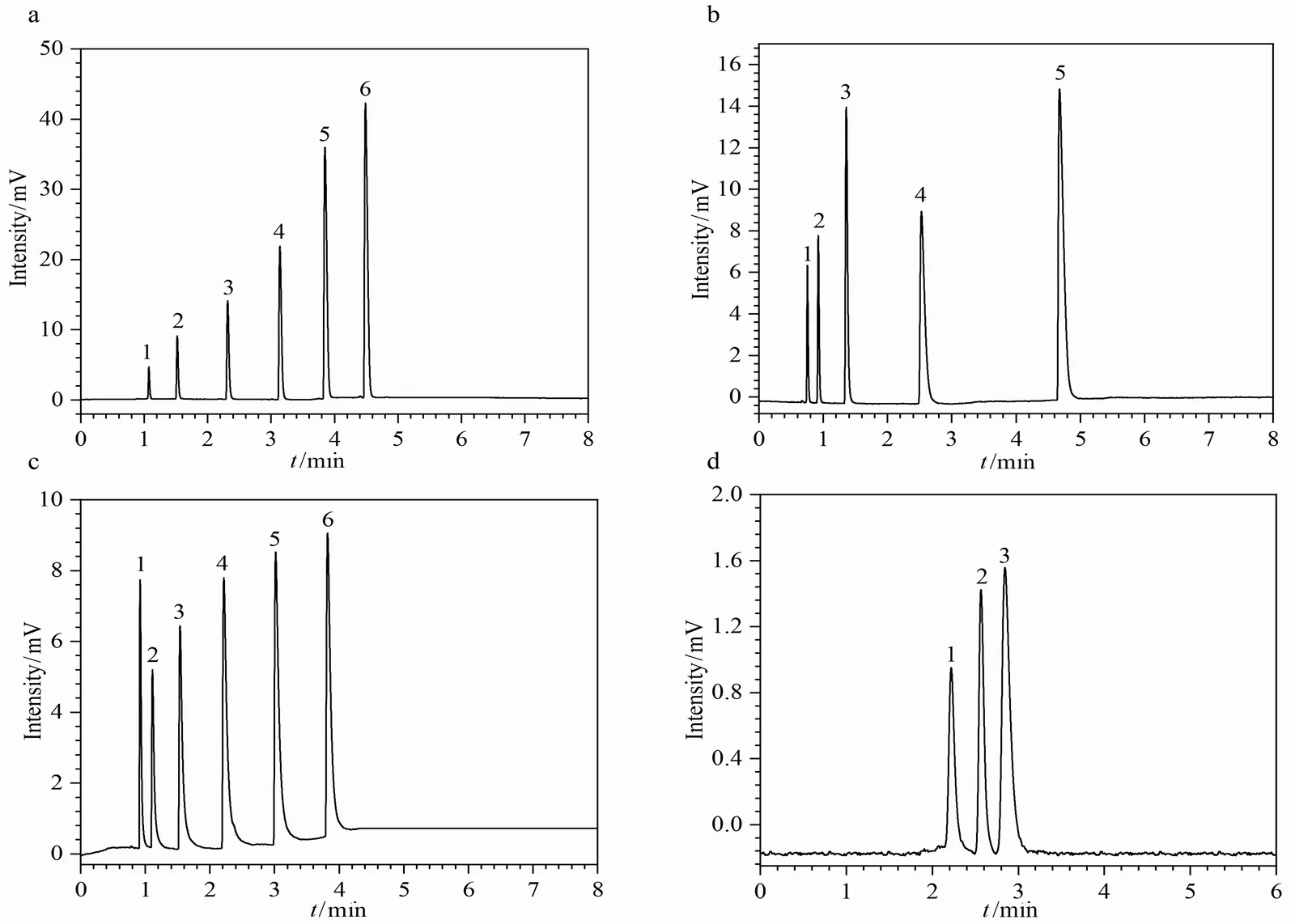
ET-COF键合毛细管柱分离（a）正构烷烃、（b）烷基苯、（c）直链脂肪酸酯以及（d）C9芳烃异构体的气相色谱图

**表1:** ET-COF键合毛细管柱分离4组化合物的分离度和理论塔板数

Analyte	Resolution （*R*_s_）	*N/*（plates/m）	Analyte	Resolution （*R*_s_）	*N/*（plates/m）
C5	/	15105	Butylbenzene	15.3	15195
C6	11.1	17493	Ethyl propanoate	/	7199
C7	15.2	25232	Ethyl butanoate	3.8	5779
C8	13.0	33571	Ethyl valerate	6.0	5533
C9	9.4	35300	Ethyl hexanoate	7.3	7305
C10	7.5	41052	Ethyl heptanoate	7.2	10141
Benzene	/	8473	Ethyl caprylate	7.0	19241
Methylbenzene	4.8	9619	Mesitylene	/	5360
Ethylbenzene	9.1	8796	Isopropylbenzene	3.0	9113
Propylbenzene	12.5	6138	Propylbenzene	2.0	4362

/ indicates no adjacent peaks and the resolution is not calculated.

本研究进一步使用同分异构体来评估ET-COF毛细管柱的分离性能，研究异孔结构（微孔择形与介孔传质协同）对分离性能的影响。C9芳烃异构体是石油化工领域重要的芳烃衍生物，在化工生产中可用作溶剂或有机合成中间体，也可作为醋酸纤维素溶剂用于纺织染料和印刷；其动力学分子直径为0.75～1.03 nm，与ET-COF的微孔孔径（1.1 nm）相近，因此被选中用于探索孔隙结构在分离中的作用。由图7d所示，ET-COF毛细管柱对3种C9芳烃异构体（正丙基苯、异丙基苯和均三甲苯）实现了基线分离，分离度均在2以上（表1），且出峰顺序为均三甲苯、异丙基苯、正丙基苯，与其分子空间构型由紧凑至细长的顺序一致，证实了1.1 nm微孔限域空间对分子形状的差异化识别作用。异孔COF的多级孔结构可提供多重尺寸排阻效应，其中微孔对小分子具有显著的尺寸排阻与择形吸附作用。

## 3 结论

本研究通过精确设计拓扑结构，成功构建了具有异孔结构的ET-COF，探究了拓扑结构对气相色谱分离性能的影响机制。其中，ET-COF基于Kagome拓扑结构，通过*π⁃π*堆积形成兼具三角形微孔与六边形介孔的双孔道体系。结构表征表明，ET-COF具有明确的异孔特征、高比表面积与良好的热稳定性，适用于色谱分离环境。在分离性能方面，采用原位生长法制备的ET-COF键合毛细管柱表现出优异的分离效能，其对于一系列同系物（正构烷烃、烷基苯、直链脂肪酸酯）以及3种C9芳烃异构体均实现了基线分离。该结果证实，异孔COF中不同尺寸孔道的协同作用可提供更丰富的相互作用界面与尺寸匹配效应，从而实现对不同尺寸与形状分子的精准识别与高效分离。本研究表明，以拓扑结构为导向理性调控COFs孔道构型与孔道尺寸分布，是提升多孔材料色谱分离性能的有效策略，突破了传统单孔COFs在分子识别与筛分能力上的局限。异孔结构带来的多重孔径协同效应，不仅为设计高选择性、高分辨色谱固定相提供了全新思路，也为复杂异构体及多组分混合物的高效分离分析提供了新型材料平台，对推动多孔晶态材料在分离科学中的理性设计与实际应用具有重要的科学启示与参考价值。未来可进一步拓展异孔固定相对手性异构体、多环芳烃异构体的分离应用，同时通过优化原位生长工艺提升柱寿命，推动其实际产业化应用。
